# Grit is associated with lower level of depression and anxiety among university students in Chiang Mai, Thailand: A cross-sectional study

**DOI:** 10.1371/journal.pone.0209121

**Published:** 2018-12-14

**Authors:** Patou Masika Musumari, Arunrat Tangmunkongvorakul, Kriengkrai Srithanaviboonchai, Teeranee Techasrivichien, S. Pilar Suguimoto, Masako Ono-Kihara, Masahiro Kihara

**Affiliations:** 1 Department of Global Health and Socio-epidemiology, Kyoto University School of Public Health, Kyoto, Japan; 2 Research Institute for Health Sciences, Chiang Mai University, Chiang Mai, Thailand; 3 Faculty of Medicine, Chiang Mai University, Chiang Mai, Thailand; 4 Center for Medical Education, Graduate School of Medicine, Kyoto University, Kyoto, Japan; Chiba Daigaku, JAPAN

## Abstract

**Background:**

Depression and anxiety symptoms are prevalent among university students in both developed and developing settings. Recently, grit, defined as perseverance and passion for long-term goals, has emerged as an indicator of success and well-being. However, the relationship between grit and poor mental health outcomes among university students is largely unknown. The current study investigates the relationship of grit with depression and anxiety among university students in Chiang Mai, Thailand.

**Methods:**

This cross-sectional study was conducted from January to March 2018 among university students aged 18–24 years from Chiang Mai University, the first largest university in Chiang Mai Province. Depression and anxiety were assessed with the Patient Health Questionnaire (PHQ-9) and the Generalized Anxiety Disorder (GAD-7) Scales, respectively. Grit was measured using the 8-item Short Grit Scale (GRIT-S). Grit scores were grouped into three categories: low (below the 25^th^ percentile); average (from the 25^th^ to the 75^th^ percentiles); and high (above the 75^th^ percentile). The other covariates included variables such self-esteem and socio-demographic variables.

**Results:**

Of the 800 participants included in the study, 405 (50.6%) were female and 395 (49.4%) were male. Respectively 21.4% and 7.8% of the participants had depression and clinical symptoms of GAD. Increasing levels of grit negatively correlated with PHQ-9 and GAD-7 scores. Participants with high level of grit scored respectively 1.69 points (*P* <0.001) and 1.71 points (*P* < 0.001) lower on the PHQ-9 scale and GAD-7 scores. Similarly, self-esteem was negatively associated with PHQ-9 and GAD-7 scores.

**Conclusion:**

Our findings highlight the negative correlation of grit with poor mental health outcomes, particularly depression and anxiety. Interventions designed to improve grit could play an essential role in the prevention of adverse mental health outcomes among university students.

## Introduction

The risk of mental health disorders, including psychological distress (depression and/or anxiety) is particularly high among university students across high-, middle-, and low-income countries [1–5]. In Thailand, the prevalence of current major depressive episodes in the general population was found to be 2.4%, based on the results from the Epidemiology of Mental Disorders National Survey 2008 [6]. An earlier survey of the general population in 2003 found a prevalence of 3.2% for major depressive disorder and 1.9% for anxiety disorder [7]. The prevalence of depression among university students in Thailand has ranged from 19% to 50% [8–12], and that of anxiety from 26% to 69% [12, 13].

University students face unique circumstances which often constitute psychosocial stressors and increase their risks of depression and anxiety. These include for example the academic stress, the anxiety for planning future careers, and also challenges associated with role transitions, such as independent living with reduced adult supervision [5, 14, 15]. The risk factors of mental disorders among university students are multiple. These include, but are not limited to, demographic and social factors (e.g. lower socio-economic status, female gender, lack of social support, lack of religiosity/spirituality), stressful or traumatic life events (e.g. loss of significant ones), psychosocial factors (e.g. substance use), and psychological factors (self-esteem, connectedness to parents, school, and friends) [1, 2, 5, 16–18]. Depression among university students is associated with a range of proximal and distal adverse social and health outcomes, such as poor academic and work performance, as well as suicide ideation and/or suicide attempts [19–22]. Therefore, all of this makes it a priority to prevent and treat mental health problems in this particular population.

Recently, grit (defined as perseverance and passion for long-term goals)[23], has received considerable attention as an independent and significant predictor of success across diverse population groups, even after controlling for influential traits, such as Intelligence Quotient (IQ) and social intelligence. This is a relatively novel construct that encapsulates two important components; namely the consistency of interest in long-term goals and persistence of effort in pursuing those long-term goals. Grit has been linked to positive educational and work-related outcomes [23, 24].

There is an increasing amount of evidence showing that grit is associated with various indicators of well-being outcomes (e.g. life satisfaction, meaning in life, psychological well-being) and quality of life [25–29]. Therefore, this is suggestive of the possibility that grit could have protective effects against adverse mental health outcomes. Gritty people are likely to be less prone to adverse mental health outcomes, due to their propensity to experience obstacles and failures in a positive way. This relation between grit and mental health outcomes was demonstrated in three recent studies, which have all employed path analysis and have document a negative relationship between grit and mental health outcomes among university students in China [30], college students with a chronic medical condition in the United States [31], and high school students in the Philippines [32]. In the latter study [32], the authors found “meaning of life” to be a mediating factor of this association, and suggested that “gritty” people are likely to be less susceptible to depression, because they are more likely to realize that life is meaningful, and so they tend to focus on maintaining perseverance and passion to achieve their long-term goals, rather than on focusing on negative aspects of life [32]. However, studies of this kind, linking grit to mental health outcomes, are remarkably scarce. Interventions aiming to improve grit could be a promising pathway for the prevention of depression and anxiety among university students. Therefore, it is critical to build evidence on the association between grit with mental health problems (depression and anxiety), in order to further research in this direction.

The current study addresses this gap in literature by investigating the association of grit with depression and anxiety among university students in Chiang Mai, Thailand.

## Methods

### Study design, participants and setting

This study employed a quantitative cross-sectional design. Data were collected from January to March 2018. The target population consisted of male and female university students aged 18–24 years, currently enrolled in Chiang Mai University, the first largest university in Chiang Mai Province, Thailand. As of December 2017, Chiang Mai University had a total of 29,035 students (10,956 males and 18,079 females).

The sample size was calculated separately for male and female students using the Krejcie and Morgan’ formula [33]. The sample size was estimated at 358.73 for male students and 368.31 female students based on the estimated population size of 10,956 for male students and 18,079 for female students, a 5% margin of error, the proportion of the population with a given interested issue sets at 0.5 (because this would provide the maximum sample size), and the chi-square value for one degree of freedom at 95% confidence interval (3.841). After adjustment for a possible 10% of non-response, the required sample size was calculated to be 395 for male students and 405 for female students, giving a total sample size of 800.

Chiang Mai University consists of twenty faculties and one college. The recruitment process included the following steps. Firstly, we grouped the faculties into three main areas: (a) Health Sciences (six faculties); (b) Science and Technology (five faculties and one college); and (c) Humanities and Social Sciences (nine faculties). Secondly, we randomly selected 4 faculties from each of the main areas. A total of 12 faculties were selected: i) Health Science area (faculties of Medicine, Dentistry, Pharmacy, and Associated Medical Sciences); ii) Science and Technology area (faculties of Engineering, Architecture, Science, and Agro-Industry); and iii) Humanities and Social Sciences area (faculties of Social Science, Education, Economic, and Political Science and Public Administration). The years of study were 1 to 6^th^ year for the faculties of Medicine, Dentistry and Pharmacy, and 1 to 4^th^ year for the rest of the faculties. Thirdly, the selected faculties were stratified by year of study (four groups: 1^st^ year, second year, third year, and fourth year and over) and by gender (male and female). Lastly, students in each of the stratum were recruited using the convenience sampling method until the intended sample size for each of the stratum was reached. The number of students recruited in each of the stratum was proportional to the size of the stratum. S1 Table provides information on the population and sample size per stratum.

### Data collection

The field research team consisted of university graduates in sociology who were trained in quantitative research methods. The participants were asked to complete the questionnaire anonymously through the Computer Assisted Self-Interviews (CASI). The structured questionnaire covered the following areas: (a) socio-economic background; (b) recreational activities; (c) smartphone and social media use; (d) intimate relationships, sexual identity and experience; (e) sexually transmitted diseases (STDs) pregnancy, abortion; and birth control; and (f) mental health.

## Measurement

### Outcomes of the study

#### Depression

Depression was assessed using the Thai version of the Patient Health Questionnaire (PHQ-9) [34]. The PHQ-9 consists of nine items, in which respondents indicate on a 4-point Likert scale (ranging from “Not at all” = 0 to “Nearly every day” = 3) how often they have experienced any of the problems over the past two weeks. The total possible score ranges from 0 to 27, with higher scores indicating more severe depression. Some sample items from the scale are “Feeling down, depressed, or hopeless” and “Poor appetite or overeating”. PHQ scores ≥ 10 had a sensitivity of 88% and a specificity of 88% for major depression [35]. In the current study, one item of the PHQ-9 (“Little interest or pleasure in doing things”) was inadvertently omitted during the Thai questionnaire preparation process. Therefore, the scores were calculated based on the remaining eight items of the PHQ-9. Consequently, the calculated prevalence, based on the cutoff point of ≥ 10 is an underestimate of the prevalence of depression in this sample. The observed Cronbach’s alpha (based on the eight items) in our sample was 0.85, suggesting a high level of internal consistency.

#### Anxiety

The Generalized Anxiety Disorder-7 (GAD-7) was used to measure symptoms of generalized anxiety disorders (GAD) [36]. The scale consists of seven items which correspond to the DSM-IV symptom criteria for GAD. Respondents are asked to indicate on a 4-point scale (ranging from “Not at all” = 0 to “Nearly every day” = 3) how often they have been bothered by any of the listed problems over the past two weeks. Some sample items include “Feeling nervous, anxious or on edge;” “Trouble relaxing;” and “Becoming easily annoyed or irritable”. The total possible score ranges from 0 to 21. Higher scores indicate more severe symptoms of GAD. In addition, the cut-off score of 10 ≥ is used to identify clinical cases of GAD [36]. The scale showed a high degree of internal consistency in our sample with Cronbach’s alpha = 0.89.

#### Primary independent variable

The primary independent variable was “grit”, measured with the 8-item Short GRIT Scale (GRIT-S) [24]. The scale consists of items which assess a respondent’s perseverance of effort (e.g., “I am a hard worker;” “Setbacks don’t discourage me”). It also included some reverse-scored items that measure consistent pursuit of passionate interest (e.g., “New ideas and projects sometimes distract me from previous ones”) using a five-point Likert scale, ranging from 1 = “Not like me at all” to 5 = “Very much like me”. The scores from all of the items are averaged to get an overall “grit score”. The total possible score ranges from 1 (“Not at all gritty”) to 5 (“Extremely gritty”). The scale showed a moderate degree of internal consistency, with Cronbach’s alpha = 0.69. Based on the distribution of scores, the level of grit among the participants was grouped into three categories: i) “Low” for scores below the 25^th^ percentile; ii) “Average” for scores ranging from the 25^th^ to the 75^th^ percentile; and iii) “High” for scores above the 75^th^ percentile. Similar categorization was applied to the self-esteem scores and the social-psychological well-being scores.

#### Other covariates

**Self-esteem:** This was assessed using the Rosenberg Self-Esteem Scale, a 10-item scale of global self-worth, which measures both positive and negative feelings (reverse-scored items) about the self on a 4-point scale, ranging from “Strongly agree” to “Strongly disagree”. Higher scores indicate higher self-esteem [37]. The observed Cronbach’s alpha in this sample was 0.83.

**Social-psychological well-being:** This was assessed with the Flourishing Scale (FS) which consists of eight items measuring a respondent’s self-perceived success in important areas, such as relationships, self-esteem, purpose, and optimism [38]. In this study, we used a 5-point Likert scale, ranging from “Strong disagreement” = 1 to “Strong agreement” = 5 (total score range 8–40). A high score indicates a person with many psychological resources and strengths. The observed Cronbach’s alpha was 0.89 indicating a high level of internal consistency.

**The other variables** included 1) Socio-demographic characteristics and parental variables (age, gender, parents’ marital status, parents’ level of education, family household income, perceived financial situation, living situation, grade average point (GPA), sexual identity); 2) connectedness variables (frequency of talking with parents; satisfaction with relationship with mother, father, and friends); 3) substance use (alcohol consumption; tobacco smoking); and 4) experience in the past year of i) self-injury (bit oneself; scratched one’s self; pulled one’s hair; interfered with wound healing; punched or banged an object to hurt one’s self); ii) being physically abused; iii) being emotionally abused; iii) striking or physically injuring anyone; iv) seriously thinking about attempting suicide; v) making a plan to attempt suicide; and vi) attempting suicide.

### Ethics statement

This study received ethical approval from the Human Experimentation Committee at the Research Institute for Health Sciences of Chiang Mai University (Certificate of Ethical Clearance No. 61/2517). The participants were first informed about the objectives of the study; about their roles; about their rights to give or not to give any information during the interview; about the confidentiality of the personal data; and about the way that the findings of the study would be presented. The participants provided their written informed consent prior to participating in the study.

### Statistical analysis

The analysis was performed using SPSS 17 (PASW) for Windows (SPSS Inc., Chicago, Illinois, USA). Univariate analysis was conducted to obtain descriptive statistics, including percentage, mean, and standard deviation (SD) of the selected variables. Differences in the mean scores of PHQ-9 and GAD-7 across categorical independent variables were assessed using t-test, ANOVA, or Kruskal-Wallis H test as appropriate.

Multiple linear regression was performed to assess the association of grit, self-esteem and other covariates with PHQ-9 and GAD-7 scores. All of the variables that were statistically significant at *p* value ≤ 0.10 in the bivariate analysis were included in the adjusted models. The variables “age”, “gender”, “father’s education level”, and “mother’s education level” were included in the model, regardless of the significance level, because of their epidemiological importance. The significance level for the multiple linear regression analysis was set at the *p* value < 0.05. There was no evidence of multicollinearity.

## Results

### Characteristics of participants

Table 1 presents the general characteristics of the participants. Out of the 800 participants included in the study, 405 (50.6%) were female and 395 (49.4%) were male. Among these participants, 23.5% were in their first year of university, while 43.4% were in their second or third years, and 33.1% in their fourth through sixth years. Slightly over half of them (52.5%) were 21 years of age and above, and most (73.4%) had parents who were either married or lived together (73.4%). Fifty-three percent of the participants lived in households with monthly incomes between 10,000 and 44,900 Thai Baht (THB) (1USD = 35THB), with most perceiving that the financial situation of their household was not a problem (62.1%). Over half of the participants had a father (56.5%) and/or a mother (54.1%) with a university or college degree. The majority of the participants reported being heterosexual (82.0%), while the remaining (18.0%) identified themselves as lesbian, gay, bisexual, or transgender (LGBT).

**Table 1:** Participants’ general characteristics and bivariate analysis with PHQ-9 and GAG-7.

	n	%	PHQ-9		GAD-7	
			(mean±SD)	p-value	(mean±SD)	p-value
**Gender**				0.316		0.342
Male	395	49.4	6.57±4.39		3.65±3.73	
Female	405	50.6	6.74±4.53		3.91±3.78	
**Age**				0.465		0.752
≤ 20 years	380	47.5	6.78±4.66		3.74±3.79	
>20 years	420	52.5	6.55±4.27		3.82±3.73	
**Education level**				0.691		0.166
1^st^ year	188	23.5	6.46±4.56		51.8±6.3	
2-3^rd^ year	347	43.4	6.80±4.39		53.2±6.5	
4-6^th^ year	265	33.1	6.60±4.47		54.4±5.6	
**Marital status of parents**				0.291		0.530
Divorced/separated	137	17.1	7.04±5.04		3.76±4.06	
Married/live together	587	73.4	6.51± 4.32		3.73±3.56	
One/both passed away	75	9.4	7.12±4.41		4.25±4.60	
*Missing values*	*1*	*0*.*1*				
**Father’s highest level of education**				0.012		0.104
Primary education or less	112	14.0	7.62±4.90		4.37±4.42	
Secondary/high school	201	25.1	6.41±3.71		3.42±3.43	
College/university	451	56.5	6.43±4.66		3.73±3.73	
Don’t know	36	4.5	7.75±3.72		4.55±3.37	
**Mother’s highest level of education**				0.193		0.063
Primary education or less	148	18.5	7.01±4.36		4.29±4.05	
Secondary/high school	207	25.9	6.75±4.27		3.32±3.76	
College/university	433	54.1	6.55±4.59		3.86±3.62	
Don’t know	12	1.5	4.25±3.46		2.58±4.18	
**Household income**				0.194		0.991
< 10,000	63	7.9	6.68±4.41		3.79±3.73	
10,000–44,999	418	52.3	6.90±4.61		3.76±4.00	
≥ 50,000	294	36.8	6.23±4.17		3.83±3.42	
Don’t know	25	3.1	7.44±5.06		3.64±3.67	
**Perceived financial status**				0.006		0.365
Financial struggle/it’s tight	303	37.9	7.21±4.05		3.94±4.00	
No financial problems	497	62.1	6.32±4.40		3.69±3.60	
**Currently live with**				0.070		0.394
Family members	368	46.0	6.27±4.37		3.59±3.70	
Friends	249	31.1	7.05±4.59		3.98±3.98	
Alone	183	22.9	6.90±4.40		3.90±3.56	
**GPA**				0.120		0.939
< 2	43	5.4	7.42±5.15		3.40±3.62	
2–2.99	425	53.1	6.90±4.69		4.04±3.78	
≥ 3	332	41.5	6.24±4.01		3.71±3.80	
**Sexual identity**				<0.001		0.045
Heterosexual	656	82.0	6.35±4.32		3.66±3.69	
LGBT	144	18.0	8.03±4.82		4.35±4.01	

PHQ-9: Patient Health Questionnaire-9; GAD-7: Generalized Anxiety Disorder-7; GPA: Grade Point Average; LGBT: lesbian, gay, bisexual, or transgender

The average level of grit scores was 3.22 (SD = 0.49). Among the participants, 206 (25.8%) were classified as having low levels of grit, while 402 (50.2%) had average levels, and 192 (24.0%) had high levels. As for self-esteem, 130 (16.3%) of the participants had low levels, while 508 (63.5%) had average levels, and 162 (20.3%) had high levels. (Table 2)

**Table 2:** Bivariate association of psychological variables, connectedness, and suicidality with depression and anxiety.

	n	%	PHQ-9		GAD-7	
			(mean±SD)	p-value	(mean±SD)	p-value
**GRIT (mean±SD)**	3.22±0.49			<0.001		<0.001
Low (< 2.88)	206	25.8	9.21±4.86		5.74±4.39	
Average (2.88–3.50)	402	50.2	6.37±4.12		3.59±3.48	
High (> 3.50)	192	24.0	4.51±3.22		2.08±2.40	
**Self-esteem (mean±SD)**	18.17±4.18			<0.001		<0.001
Low (< 15)	130	16.3	11.07±5.10		7.07±4.59	
Average (15–21)	508	63.5	6.40±3.81		3.53±3.35	
High (> 21)	162	20.3	3.90±2.92		1.94±2.34	
**PWB (mean±SD)**	33.28±4.90			<0.001		<0.001
Low (< 30)	167	20.9	9.11±5.12		5.53±4.44	
Average (30–37)	453	56.6	6.50±4.14		3.80±5.59	
High (>37)	180	22.5	4.77±3.46		2.11±2.52	
**History of self injury**				<0.001		<0.001
No	705	88.1	6.28±4.26		3.40±3.46	
Yes	95	11.9	9.41±4.95		6.57±4.59	
**Alcohol use (past 1 month)**				0.607		0.625
No	350	43.8	6.56±4.45		3.86±3.75	
Yes	450	56.3	6.73±4.46		3.72±3.76	
**Smoking (past 1 month)**				0.241		0.188
No	704	88.0	6.59±4.37		3.72±3.71	
Yes	96	12.0	7.16±5.06		4.26±4.08	
**How often do you talk to your parents?**				<0.001		<0.001
Not at all/Not often	215	26.9	8.21±5.13		4.93±4.45	
Neutral	88	11.0	7.17±4.62		3.77±3.90	
Regularly/Often	495	61.9	5.87±3.89		3.27±3.25	
*Missing values*	*2*	*0*.*3*				
**Perceived satisfaction with relation with father**				<0.001		0.001
Dissatisfied	32	4.0	10.0±5.98		6.25±5.15	
Neutral	109	13.6	7.73±4.65		4.25±3.96	
Satisfied	616	77.0	6.21±4.18		3.48±3.45	
*Missing values*	*43*	*5*.*4*				
**Perceived satisfaction with relation with mother**				<0.001		<0.001
Dissatisfied	15	1.9	12.13±6.94		8.46±6.23	
Neutral	65	8.1	8.46±5.45		5.30±4.72	
Satisfied	711	88.9	6.37±4.18		3.54±3.49	
*Missing values*	*9*	*1*.*1*				
**Perceived satisfaction with relation with friends**				<0.001		<0.001
Dissatisfied	24	3.0	11.08±6.08		6.95±5.76	
Neutral	98	12.3	9.20±4.61		5.72±3.86	
Satisfied	678	84.8	6.13±4.15		3.39±3.51	
**Has physically assaulted someone in the past year**				<0.001		0.003
No	745	93.1	6.50±4.42		3.67±3.73	
Yes	55	6.9	8.73±4.43		5.23±3.83	
**Has been emotionally abused in the past year**				<0.001		<0.001
No	447	55.9	5.55±4.06		2.78±3.28	
Yes	353	44.1	8.05±4.55		5.05±3.94	
**Has been physically abused in the past year**				<0.001		<0.001
No	755	94.4	6.50±4.40		3.65±3.71	
Yes	45	5.6	9.33±4.52		6.04±3.84	
**Has seriously thought about attempting suicide in the past year**				<0.001		<0.001
No	769	96.1	6.39±4.15		3.55±3.47	
Yes	31	3.9	13.26±6.46		9.41±5.78	
**Has made a plan for attempting suicide in the past year**				<0.001		<0.001
No	773	96.6	6.45±4.24		3.61±3.57	
Yes	27	3.4	12.67±6.08		8.74±5.44	
**Has attempted suicide in the past year**				<0.001		0.001
No	790	98.8	6.53±4.30		3.68±3.61	
Yes	10	1.3	16.40±6.04		12.1±5.91	
History of using mental health services						
No	751	93.9	6.42±4.23	<0.001	3.61±3.55	
Yes	49	6.1	10.27±6.15	0.001	6.46±5.45	

PHQ-9: Patient Health Questionnaire-9; GAD-7: Generalized Anxiety Disorder-7; PWB: Psychological Well-Being

### Prevalence of depression and generalized anxiety disorder

The prevalence of major depression in this study has been calculated based on eight items of the PHQ-9. Therefore, the prevalence in this study is an underestimate of the real prevalence in the sample. Based on the cutoff point of ≥ 10, 171 (21.4%) were classified as having major depression. In terms of assessment of anxiety, 62 (7.8%) of the participants were categorized as having clinical GAD (based on the GAD-7 cutoff score of ≥ 10).

### Factors associated with depression and GAD

Tables 1 and 2 show variables that were found to be significantly associated with higher/lower PHQ-9 and GAD-7 scores in the bivariate analysis. In the adjusted models (Table 3), we found that those participants who had high levels of grit had PHQ-9 scores and GAD-7 scores that were on average, respectively 1.71 points (*P* < 0.001) and 1.73 points (*P* < 0.001) lower than in participants with low levels of grit. The same pattern was noted when comparing participants with average levels of grit to those with low levels of grit. Similarly, participants with high and average levels of self-esteem had PHQ-9 (*B* = -4.13; *P* < 0.001 for high self-esteem) and GAD-7 (*B* = -2.43; *P <* 0.001 for high self-esteem) scores that were, on average lower compared to those of the participants with low self-esteem.

**Table 3:** Multivariable analysis of factors associated with depression and anxiety.

	PHQ-9			GAD-7		
	B	95% CI	p-value	B	95% CI	p-value
**GRIT** High (vs low)	-1.713	-2.551; -0.875	<0.001	-1.737	-2.470; -1.004	<0.001
**GRIT** Average (vs low)	-1.157	-1.806; -0.509	<0.001	-1.018	-1.585; -0.450	<0.001
**Self-esteem** High (vs low)	-4.134	-5.143; -3.125	<0.001	-2.434	-3.316; -1.551	<0.001
**Self-esteem** Average (vs low)	-2.677	-3.472; -1.881	<0.001	-1.871	-2.567; -1.175	<0.001
**PWB** High (vs low)	-0.860	-1.750; 0.030	0.058	-0.742	-1.521; 0.037	0.062
**PWB** Average (vs low)	-0.359	-1.080; 0.362	0.329	0.059	-0.572; 0.690	0.853
**Gender** Female (vs male)	0.461	-0.059; 0.980	0.082	0.480	0.025; 0.934	0.039
**Age years** >20 (vs ≤ 20)	-0.550	-1.292; 0.193	0.147	-0.067	-0.717; 0.583	0.840
**Education level**						
2-3^rd^ year (vs 1^st^ year)	0.539	-0.185; 1.263	0.144	0.492	-0.141; 1.126	0.127
4-6^th^ year (vs 1^st^ year)	0.639	-0.358; 1.637	0.209	0.179	-0.694; 1.051	0.688
**Father’s highest level of education**						
Primary education or less (ref)						
Secondary/high school	-1.074	-1.986; -0.162	0.021	-0.607	-1.405; 0.191	0.136
College/university	-1.190	-2.168; -0.212	0.017	-0.520	-1.376; 0.336	0.233
Don’t know	-0.889	-2.384; 0.607	0.244	-0.415	-1.723; 0.893	0.534
**Mother’s highest level of education**						
Primary education or less (ref)						
Secondary/high school	0.729	-0.109; 1.566	0.088	-0.341	-1.074; 0.392	0.361
College/university	0.534	-0.357; 1.426	0.240	-0.073	-0.853; 0.707	0.855
Don’t know	-2.777	-5.155; -0.398	0.022	-2.096	-4.177; -0.015	0.048
**Perceived financial status**						
Financial struggle/it’s tight (ref)						
No financial problems	0.078	-0.0475; 0.630	0.783	0.402	-0.082; 0.885	0.103
**Currently live with**						
Family members (vs alone)	-0.613	-1.251; 0.024	0.059	-0.281	-0.838; 0.277	0.324
Friends (vs alone)	-0.389	-1.092; 0.314	0.277	-0.330	-0.945; 0.285	0.292
**GPA** < 2 (vs ≥ 3)	0.312	-0.888; 1.512	0.610	-0.563	-1.613; 0.486	0.292
**GPA** 2–2.99 (≥ 3)	0.028	-0.505; 0.560	0.918	-0.189	-0.655; 0.277	0.426
**LGBT** (vs heterosexual)	0.557	-0.106; 1.221	0.100	-0.173	-0.754; 0.407	0.558
**Self-injury** Yes (vs no)	0.792	-0.048; 1.631	0.065	1.372	0.638; 2.107	<0.001
**How often do you talk to your parents?**						
Regular (vs not at all/not often)	-0.835	-1.458; -0.211	0.009	-0.498	-1.044; 0.047	0.073
Neutral (vs not at all/often)	-0.344	-1.244; 0.557	0.454	-0.546	-1.334; 0.242	0.174
**Perceived satisfaction with relation with father**						
Satisfied (vs dissatisfied)	-0.742	-2.195; 0.711	0.316	-0.057	-1.328; 1.214	0.930
Neutral (vs dissatisfied)	-0.708	-2.252; 0.835	0.368	-0.527	-1.878; 0.823	0.444
**Perceived satisfaction with relation with mother**						
Satisfied (vs dissatisfied)	-0.862	-2.939; 1.216	0.416	-1.571	-3.388; 0.247	0.090
Neutral (vs dissatisfied)	-0.798	-3.013; 1.416	0.479	-0.731	-2.669; 1.206	0.459
**Perceived satisfaction with relation with friends**						
Satisfied (vs dissatisfied)	-2.444	-4.023; -0.865	0.002	-1.545	-2.927; -0.164	0.028
Neutral (vs dissatisfied)	-1.063	-2.739; 0.613	0.213	-0.469	-1.935; 0.997	0.530
**Has physically assaulted someone in the past year**						
Yes (vs no)	0.460	-0.700; 1.620	0.436	-0.445	-1.460; 0.570	0.389
**Has been emotionally abused in the past year**						
Yes (vs no)	0.977	0.446; 1.507	<0.001	1.085	0.620; 1.549	<0.001
**Has been physically abused in the past year**						
Yes (vs no)	0.830	-0.475; 2.135	0.212	1.282	0.141; 2.424	0.028
**Has seriously thought about attempting suicide in the past year**						
Yes (vs no)	1.670	-0.021; 3.360	0.053	1.565	0.086; 3.044	0.038
**Has made a plan for attempting suicide in the past year**						
Yes (vs no)	0.235	-1.477; 1.948	0.787	0.348	-1.150; 1.847	0.648
**Has attempted suicide in the past year**						
Yes (vs no)	1.774	-0.906; 4.455	0.194	1.661	-0.684; 4.006	0.165
**History of using mental health services**						
Yes (vs no)	1.659	0.531; 2.786	0.004	0.914	-0.072; 1.901	0.069

PHQ-9: Patient Health Questionnaire-9; GAD-7: Generalized Anxiety Disorder-7; PWB: Psychological well-being; LGBT: lesbian, gay, bisexual, or transgender; ref: reference group; vs: versus

In regards to connectedness, those participants who reported that they regularly or often talk to their parents had lower levels of PHQ-9. On average, their scores were 0.83 points (*P* = 0.009) lower than for those who responded that they do not often or do not talk at all to their parents. The same trend was noted for GAD-7 scores, though it did not attain statistical significance (*B* = -0.49; *P* = 0.073). In addition, participants who rated their relationship with their friends as satisfactory had PHQ-9 scores and GAD-7 scores which were lower than for those who were not satisfied (*B* = -2.44; *P* = 0.002 for PHQ-9; *B* = -1.54; *P* = 0.028 for GAD-7). The level of father’s education was also found to be related to PHQ-9 scores. Participants whose parents had college/university degrees or secondary/high school levels had PHQ-9 scores that were lower than the scores of those whose parents had only attained the level of primary education or below. We also found that being female correlated with higher level of anxiety (*B* = 0.48; *P* = 0.039).

Most variables related to suicidality and violence (either the perpetration or undergoing of violence) lost statistical significance in the adjusted model, with the exception of “history of being emotionally abused” which was associated with higher PHQ-9 and GAD-7 scores, and “ever had seriously thought about attempting suicide” which was positively correlated with higher GAD-7 scores. Having a history of self-injury in the past year was positively correlated with GAD-7 scores (*B* = 1.37; *P* < 0.001), with a similar but non-significant trend for the PHQ-9 scores (*B* = 0.79; *P* = 0.065). On the other hand, having a history of using mental health services was positively correlated with PHQ-9 scores (*B* = 1.65; *P* = 0.004), with a similar but non-significant trend for the GAD-7 scores (*B* = 0.91; *P* = 0.069).

The models explained respectively 41.5% and 37.0% of the variation in PHQ-9 and GAD-7 scores.

## Discussion

This is one of the first studies to report the association of grit with mental health outcomes among the population of university students. One of the strengths of this study is that it included other psychological constructs (e.g. self-esteem; social-psychological well-being; etc.) and variables which could potentially either confound or mediate the association between grit and mental health outcomes (depression and anxiety).

Most studies on grit have focused on its role as a predictor of academic and life success, and on its various well-being outcomes (e.g., life satisfaction, meaning in life, and psychological well-being)[23–27]. Only very limited research has examined the link between grit and mental health outcomes. However, emerging evidence overall indicates a negative correlation between grit and mental health outcomes. A recent study among university students in China revealed a negative relationship between one component of grit (consistency of interests) and depression, anxiety and stress. In the same study, however, it was found that perseverance of effort (another component of grit) was associated with depression but not with anxiety and stress [30]. Similarly, another recent study using path analysis found that grit was negatively associated with depression in a sample of high school students in the Philippines [32]. This current study strengthens the existing evidence by showing that higher levels of grit are associated with lower levels of depression and anxiety as assessed by PHQ-9 and GAD-7 respectively. Although we did not explore the pathway(s) through which grit is related to depression and anxiety, the previous study identified “meaning of life” as a factor mediating the association of grit with depression [32]. Gritty individuals are more likely to realize that life is meaningful; therefore, they tend to maintain perseverance and passion in order to achieve their long-term goals. In turn, individuals who have found meaning in their lives are less likely to be depressed [32].

University students are particularly at high-risk for mental health problems. The finding that higher grit is related to lower levels of depression and anxiety indicates that cultivating grit is likely to be a promising channel for the prevention of mental health problems among university students. Studies which have investigated the influential factors of grit are remarkably few. The growth mindset, which refers to the belief that a person’s intelligence is malleable and can be cultivated through effort and hard work [39], has received considerable attention in past years, and evidence on its positive effects on grit has started to accrue [39–42]. Operationalization of the growth mindset and other potential influential factors of grit within a training or educational program, and testing of their effect on grit should be a priority in future research. In addition, measuring grit could also be a strategy to identify students at greater risk for poor mental health.

In consistence with previous studies among university students and other population groups [43–45], we found that higher levels of self-esteem were associated with lower levels of depression and anxiety. In this study, self-esteem had the strongest correlation with both depression and anxiety, suggesting that improvement of self-esteem could be an effective strategy for the prevention of mental health problems among university students.

We also found evidence that higher paternal education level was associated with lower levels of depression. However, this association was not statistically significant for maternal education, although previous studies have mostly reported on the positive impacts of maternal education on the health, psychosocial, and cognitive outcomes of the children [46, 47]. In a recent study from China [48], low parental educational level was associated with depression in college students, but the authors did not discriminate between the effect of maternal and paternal education. A longitudinal population-based study in Canada revealed that low maternal education, but not low paternal education, was associated with increased risk of major depressive episodes in early adulthood [49]. The authors suggested that the parenting skills of mothers, given their usually prominent role in childrearing, or the social learning that takes place in terms of teaching coping skills, might have stronger impacts on the risk of depression for their children. In regarding to our contrasting findings, mothers in Thailand may have the same extensive role in childrearing role. However, the type of parenting style chosen could largely depend on the father. Future research should, therefore, examine the possible mediating role of parenting style in the relationship between parental education and depression in children.

In this study, it was found that having good relationships with friends was associated with lower levels of depression and anxiety. In addition, in the bivariate analysis, all the variables which were indicative of good connectedness with parents (satisfaction with relationship with father, mother, and regularly/often talking with parents) were all negatively associated with both depression and anxiety. However, only “regularly/often to talking with parents” remained statistically significant in the adjusted models. These results corroborate previous research showing that family and peer connectedness (e.g. feelings of warmth, love, and caring from parents and other family members and friends) is negatively associated with depression and anxiety among young people. Young people who feel more connected to their families and friends are likely to have a greater sense of belonging and being supported, which in turn could act as a buffer against depression and other psychological stress [50, 51]. Thus, the results of this current study support strategies which enhance connectedness to family and friends, as a way of reducing the risk of depression and anxiety among university students, and other groups of youth in general.

This study has a number of limitations. Firstly, no causal inference can be drawn from the documented associations due to the cross-sectional design of the study. Secondly, although participants were recruited from the largest university in Chiang Mai, our findings might not be generalizable to students from other universities in Chiang Mai and to other settings in Thailand given the potential differences in mental health problems in and services available for students across different universities. Thirdly, one significant limitation of this study was that one item was missed in our assessment of depression, the PHQ-9. Although the 8 items which were included demonstrated a high level of internal consistency (Cronbach’s alpha = 0.85), it is unclear to what extent the incompleteness of the PHQ-9 scale could influence the results of the study. In addition, PHQ-9 cut-off point of ≥ 10, which has been associated with the clinical diagnosis of major depression was based on the full PHQ-9 scale [35]. Therefore, using the same cut-off threshold with smaller number of items will result in the underestimation of the real prevalence of depression among this population of students. For this reason, we could not dichotomize the PHQ-9 scores, based on the cut-off point of ≥ 10 in order to estimate the effect size of the risk of having depression across different levels of grit, using logistic regression analysis. Lastly, it is important to note that other concurrent life stressors likely to affect the mental health status of students were not assessed in this particular study. These include, for example, negative life events, health problems, and interpersonal difficulties. Future research would do well by including such variables in the model, so that the extent to which grit is related to mental health outcomes can be comprehensively investigated.

Overall, this study presents novel evidence demonstrating grit as an independent predictor of mental health outcomes among a population of university students. The negative correlation of grit with depression and anxiety suggests that interventions designed to improve grit could play an essential role in the prevention of adverse mental health outcomes among university students. Ideally, the prevention of depression and anxiety should be holistic, by way of concurrently addressing the other influencing factors of mental health, such as self-esteem and connectedness to family members and friends.

## Supporting information

S1 Dataset Dataset of the study. (SAV)

S1 Table Information on the population and sample size. (DOCX)
