# Supplementary material for: Grit is associated with lower level of depression and anxiety among university students in Chiang Mai, Thailand: A cross-sectional study
Source: PLoS One. 2018 Dec 14;13(12):e0209121. doi: 10.1371/journal.pone.0209121 (PMC6294431; doi:10.1371/journal.pone.0209121)
Supplement: S1 Table — (DOCX) [file pone.0209121.s002.docx]

**S1 Table. Information on the population and sample size**

|  | **Population size** | | | | | | | | | | **Sample** | **Sample size** | | | | | | | | | |
| --- | --- | --- | --- | --- | --- | --- | --- | --- | --- | --- | --- | --- | --- | --- | --- | --- | --- | --- | --- | --- | --- |
|  | Year 1 | | Year 2 | | Year 3 | | Year 4 or more | | Total | |  | Year 1 | | Year 2 | | Year 3 | | Year 4 or more | | Total | |
|  | M | F | M | F | M | F | M | F | M | F |  | M | F | M | F | M | F | M | F | M | F |
| **Sciences and Technology (5 faculties and 1 collage)**  Faculty of Science  Engineer  Agriculture  Industry  Architect  College of media and technology | 213  704  137  69  46  122 | 364  337  226  234  66  103 | 190  588  134  74  32  99 | 284  216  235  244  67  84 | 191  540  110  60  33  111 | 272  205  249  238  51  77 | 279  762  151  81  67  232 | 309  202  234  258  91  151 | 873  2594  532  284  178  564 | 1229  960  944  974  275  415 | Science  Engineer  Agro-Industry  Architect | 12  39  4  3 | 16  15  10  3 | 10  32  4  2 | 13  10  11  3 | 10  30  3  2 | 12  9  11  2 | 16  42  5  4 | 14  9  11  4 | 48  143  16  11 | 55  43  43  12 |
| Total (Sciences and Technology) | 1291 | 1330 | 1117 | 1130 | 1045 | 1092 | 1572 | 1245 | 5025 | 4797 | Total | 58 | 44 | 48 | 37 | 45 | 34 | 67 | 38 | 218 | 153 |
| **Health Sciences (6 faculties)**  Faculty of Medicine  Dentistry  Pharmacy  Associated Medical Sciences  Nursing  Veterinary | 116  26  51  76  52  19 | 137  38  105  245  581  48 | 120  28  41  74  53  13 | 110  48  93  226  587  39 | 137  31  35  82  61  19 | 103  48  91  215  526  39 | 371  113  131  92  53  61 | 379  193  289  230  615  119 | 744  198  258  324  219  112 | 729  327  578  916  2309  245 | Medicine  Dentistry  Pharmacy  Associated Medical | 6  2  3  4 | 6  2  5  11 | 7  2  2  4 | 5  2  4  10 | 7  2  2  5 | 5  2  4  10 | 21  6  7  5 | 17  9  13  10 | 41  12  14  18 | 33  15  26  41 |
| Total (Health Sciences) | 340 | 1154 | 329 | 1103 | 365 | 1022 | 821 | 1825 | 1865 | 5104 | Total | 15 | 24 | 15 | 21 | 16 | 21 | 39 | 49 | 85 | 115 |
| **Humanities and Social Sciences (9 faculties)**  Humanities  Education  Social Sciences  Fine Arts  Accounting and Management  Economics  Mass communication  Political Science  Law | 172  122  74  144  151  72  67  92  106 | 486  218  133  185  377  114  150  155  153 | 150  106  69  116  120  141  79  84  90 | 405  210  106  174  334  192  191  143  141 | 123  104  49  102  151  152  88  100  85 | 376  183  114  175  393  336  167  154  05 | 176  156  63  130  137  191  86  122  106 | 450  361  138  203  377  354  127  165  133 | 621  488  255  492  559  556  320  398  387 | 1717  972  491  737  1481  996  635  617  532 | Education  Social Sciences  Economics  Political Sciences | 7  4  4  5 | 10  6  5  7 | 5  4  8  5 | 9  5  9  6 | 6  3  8  5 | 8  5  15  7 | 7  3  11  7 | 16  6  16  7 | 25  14  31  22 | 43  22  45  27 |
| Total (Humanities and Social Sciences) | 1000 | 1971 | 955 | 1896 | 954 | 2003 | 1167 | 2308 | 4076 | 8178 | Total | 20 | 28 | 22 | 29 | 22 | 35 | 28 | 45 | 92 | 137 |
| **Total of Population** | **2631** | **4455** | **2401** | **4129** | **2364** | **4117** | **3560** | **5378** | 10956 | 18079 | **Total of Sample** | **93** | **96** | **85** | **87** | **83** | **90** | **134** | **132** | **395** | **405** |

Source: Registration Office, Chiang Mai University, Semester 1, Year 2017 (data on 25 October 2017)
